# Identification of Zimbabwe’s locally grown banana (*Musa* Spp.) cultivars using morphology and genome-targeted sequencing

**DOI:** 10.1186/s43141-023-00562-1

**Published:** 2023-11-14

**Authors:** Kumbirai Beaton, Allen Mazadza, Zedias Chikwambi

**Affiliations:** Department of Biotechnology, Chinhoyi University of Technology, P.Bag 7724, Chinhoyi, Zimbabwe

**Keywords:** Banana, Target sequencing, Genetic diversity, Inter transcribed spacer

## Abstract

**Background:**

Banana production is increasingly under threat due to harsh weather conditions as a result of climate change and different diseases. As such there is a need for the preservation and the characterization of the banana cultivar population for the purposes of crop improvement. The identification of collected banana germplasm in Zimbabwe was conducted based on the Inter-transcribed spacer region as well as morphology. The study was conducted with the aim of distinguishing one cultivar from another towards genetic conservation as well as banana improvement.

**Results:**

ITS 1 and ITS 4 region targeting primers were used to amplify the DNA from twelve cultivars as well as sequence. Blast results identified five *Musa* groups which are *Musa balbisiana (BB), Musa ABB, Musa AB hybrid, Musa acuminata (AAA)*, and *Musa acuminata subsp. Malaccensis (AA).* Phylogenetic analysis was done on the sequences under study and a maximum likelihood tree was generated to determine relationships between the sequences. Further identification was done using the inflorescence, bract, and male bud and fruit characteristics of each cultivar complementing the molecular evaluation.

**Conclusion:**

Genetic and morphological identification of locally grown bananas was therefore successful. An important step towards identifying pure lines suitable for breeding.

## Background

Bananas are described as herbaceous plants that can confer the aspect of a tree [1]. The plant has a pseudo-stem which is formed by the concentric assembly of leaf sheaths, crowned by a rosette of large oblong to elliptic leaves. The leaves of a banana plant are produced successively until the inflorescence is cast [2]. Being a perennial crop, banana, like all the other perennial crops, offers food security to most developing countries in the world [3]. Globally, banana (*Musa* Spp) is ranked fourth in terms of gross value production following rice, wheat, and maize [4]. Its production in Zimbabwe is ranked 57 among 132 producing countries. Banana production in Zimbabwe is constituted by both the commercial varieties and the landrace varieties. Genetic characterization in Zimbabwe has mostly targeted crops such as maize, [5], sunflowers [6], sorghum [7], sugarcane [8], tobacco [9], and wheat [10] amongst other crops. Documented characterization in bananas has been carried out on plant parasitic nematodes from Rusitu Valley cultivars but to the researchers’ knowledge, no information has been made available on banana genetics in Zimbabwe [11].

Regardless of the social, cultural, and economic significance of bananas, little is known about their organization and evolution. The *Musaceae* family, which belongs to the order *Zingiberales*, consists of three *genera Musa*, *Ensete,* and *Musella*. The traditional morpho-taxonomic classification of bananas is based on a set of morphological descriptors and basic chromosome numbers. This conventional taxonomy has been often questioned and the phylogenetic relationships within the family *Musaceae* remain subject to debate. Different types of molecular markers have been used until now to investigate the diversity of bananas at various taxonomic levels but the taxonomy and phylogenetic relationships remain unresolved.

Molecular markers have been used extensively for banana characterization which include random amplified polymorphic DNA markers (RAPD’s) with over a thousand research publications [12], restriction fragmentation length polymorphism markers (RFLPs) [13], simple sequence repeats (SSR) [14], fragment length polymorphism markers (AFLPs [15], and diversity array technology markers (DArTs) [16], each method having its own pros and cons. Genetic analysis using ribosomal DNA (rDNA) has often been used to analyze genetic variability and relationships among banana cultivars. The rDNA is the coding region of the RNA component of the ribosome which constitutes the 18S sub-unit, the 5.8S, and the large sub-unit which is the 26S. An internal transcribed spacer (ITS) separates these units [17]. The use of the ITS region in investigating phylogenetic studies has become widespread and this is mainly because the region is highly variable due to frequently occurring nucleotide polymorphisms or insertions/deletions in the sequence and is phylogenetic inference at the specific and generic levels [3]. It also has a bi-parental inheritance compared to the uniparental inheritance of chloroplast and mitochondrial markers and the intra-genomic sequence uniformity caused by active homogenization of repeat units, also known as concerted evolution. Owing to these and other varied properties, the ITS region became one of the most widely used sequences for phylogenetic inferences. ITS 1 and ITS 4 primers have been used in the identification of Truffles [6, 18], *Trichoderma* [14], amongst others.

The objective of this study was to characterize and assess the genetic relationship amongst collected accessions grown in Zimbabwe targeting the ITS regions of the accession sequences as well as morphological characterization based on selected descriptors of the accessions.

## Methods

### DNA extraction

Young leaves of 12 banana plantlets were collected from the Chinhoyi University Farm gene bank and DNA was isolated using the modified CTAB method [19]. CTAB buffer was pre-heated at 60 °C in a water bath before being added to 1 g of fresh leaf sample in a pre-heated mortar. The sample was ground and incubated at 60 °C for 30 min with occasional swirling. Sample was then cooled for 5 min and centrifuged for 1 min at 6000 × *g*. Using equal volumes, chloroform-isoamyl alcohol (Skylabs, South Africa) extraction (in the ratio 24:1, v/v) was performed once and centrifuged for 5 min at 6000 × *g*. The aqueous phase was transferred to a new tube and chloroform-isoamyl extraction was repeated until the upper phase was clear. To the aqueous phase, Proteinase K (New England *BioLabs*, U.K) was added and the mixture was incubated at 37 °C for 30 min. Sample was placed in an ice-bath and ice-cold isopropanol alcohol (Glassworld, South Africa) was slowly added down the side of the tube until white stringy DNA precipitated. The DNA-containing phase was transferred to a new tube and centrifuged at 10,000 × *g* for 5 min until a pellet was recovered. The liquid phase was decanted and the pellet was washed in 70% ethanol and dried. The pellet was dissolved in T.E buffer and DNA was ready for amplification. The genomic DNA concentration was analyzed using the BioDrop µLite 1380 machine. The µLite 0.5 mm path length was used with a dilution factor of 1.

### DNA amplification

Amplification of the genomic DNA was carried out using ITS 1 and ITS 4 primer sequences (synthesized at Inqaba Biotech, South Africa). ITS 1 5′ (TCCGTAGGTGAACCTGCGG) and 3′ (GGCGTCCAAGTGGATGCCT) and ITS 4 3′ (TCCTCCGCTTATTGATATGC) and 5′ (CGTATAGTTATTCGCCTCCT).

PCR was carried out in 25 μL reaction volumes containing One Taq DNA polymerase 2 × Master Mix with standard buffer (12.5 μL), 10 μM, forward primer (0.5 μL), 10 μM reverse primer (0.5 μL) and nuclease-free water (9.5 μL) (New England *BioLabs*, UK). An initial denaturation and enzyme activation step of 10 min at 95 °C was followed by amplification for 35 cycles at the following conditions: 30 s at 95 °C, 40 s at 60 °C, 40 s at 72 °C. A final 5-min extension at 72 °C completed the protocol and was held at 4 °C. The PCR products were separated on agarose gel (1%) in 0.5 × TBE buffer and stained with ethidium bromide for band visualization (Oxford Laboratory Reagents, India).

#### Sequencing

The ITS 1 and ITS 4 amplicons were purified and then sequenced using an ABI 3500XL Genetic Analyzer Sanger sequencing machine.

### Phylogenetic analysis

The 24 sample sequences were trimmed in Chromas v2.6.6 before being exported as fasta files. Phylogenetic and molecular evolutionary analyses were conducted using MEGA version 11 [20]. Additional ITS sequences were downloaded from NCBI for comparison with the sample sequences. Clustal W was used for sequence alignment and evolutionary analysis of the *Musa* species was analyzed using the Maximum Likelihood method and Tamura-Nei model. Bootstrapped consensus tree for the Maximum Likelihood method was obtained from 1000 replicates. Initial tree(s) for the heuristic search were obtained automatically by applying Neighbor-Join and BioNJ algorithms to a matrix of pairwise distances estimated using the Tamura-Nei model, and then selecting the topology with superior log likelihood value. Phylogenetic and molecular evolutionary analyses were conducted using MEGA version 11 [20]. The phylogenetic trees were rooted using an outgroup species *Ensete ventricosum*.

BLAST program was inferred to identify similar sequences and Tables 1 and 2 show the blast results.

**Table 1:** ITS 4 sequence blast results

Sample sequence	Blast similar sequence	Similarity %	Ambiguity %	Length (bp)	GC content
Bk01_ITS4_G04_3730XL	*Musa* hybrid cultivar AB Group	84.39	15.61	734	57.59
Bk02_ITS4_H04_3730XL	*Musa* *acuminata* subsp. *Malaccensis* *AAA Group*	99.52	0.48	733	63.57
Bk03_ITS4_A05_3730XL	*Musa* *acuminata* subsp. *Malaccensis* *AAA Group*	99.68	0.32	849	60.54
Bk04_ITS4_B05_3730XL	*Musa* *acuminata* subsp. *Malaccensis* *AAA Group*	93.50	6.5	958	57.10
Bk05_ITS4_C05_3730XL	*Musa balbisiana* B genome	93.35	6.65	781	63.00
Bk06_ITS4_D05_3730XL	*Musa balbisiana* *B genome*	99.52	0.48	630	63.97
Bk07_ITS4_E05_3730XL	*Musa* *acuminata* subsp. *Malaccensis* *AAA group*	99.84	0.16	866	61.89
Bk08_ITS4_F05_3730XL	*Musa* *acuminata* subsp. *Malaccensis* AAA Group	99.68	0.32	744	63.84
Bk09_ITS4_G05_3730XL	*Musa* *acuminata* subsp. *Malaccensis*	99.84	0.16	738	63.01
Bk10_ITS4_G02_3730XL	*Musa hybrid* *AB Group*	87.83	12.17	384	58.33
Bk11_ITS4_A06_3730XL	*Musa balbisiana* *B genome*	99.52	0.48	820	63.41
Bk12_ITS4_B06_3730XL	*Musa* *acuminata* subsp. *Malaccensis* AAA Group	99.52	0.48	796	62.94

**Table 2:** Sequence blast of ITS 1 sequences

Sample sequence	Blast similar sequence	Similarity %	Ambiguity %	Length (bp)	Gc content
Bk01_ITS1_C03_3730XL	*Musa Acuminata* AAA Group	84.32	15.68	628	60.71
Bk02_ITS1_D03_3730XL	*Musa* *acuminata* subsp. *Malaccensis* *AAA Group*	99.36	0.64	625	63.36
Bk03_ITS1_E03_3730XL	*Musa* *acuminata* subsp. *Malaccensis* *AAA Group*	99.84	0.16	627	63.48
Bk04_ITS1_F03_3730XL	*Musa* *acuminata* *AAA Group*	84.47	15.53	625	60.96
Bk05_ITS1_G03_3730XL	*Musa ABB* ABB group	99.68	0.32	622	63.99
Bk06_ITS1_H03_3730XL	*Musa ABB* *ABB Group*	99.68	0.32	626	63.74
Bk07_ITS1_A04_3730XL	*Musa* *acuminata* subsp. *Malaccensis* *AAA group*	99.84	0.16	628	63.38
Bk08_ITS1_B04_3730XL	*Musa* *acuminata* subsp. *Malaccensis* AAA Group	99.84	0.16	618	63.11
Bk09_ITS1_C04_3730XL	*Musa* *acuminata* subsp. *Malaccensis*	99.52	0.48	625	63.52
Bk10_ITS1_D04_3730XL	*Musa* *acuminata* subsp. *Malaccensis*	99.84	0.16	624	63.30
Bk11_ITS1_E04_3730XL	*Musa ABB* *ABB Group*	99.52	0.48	630	63.65
Bk12_ITS1_F04_3730XL	*Musa* *acuminata* subsp. *Malaccensis* AAA Group	98.07	1.93	625	63.52

### Morphological characterization

Morphological characterization of the *Musa* cultivars was carried out focusing on the inflorescence, bract, male flower, and the fruit [21]. Qualitative criteria were used and morphological character traits were matched against the IPB-INIBAP Banana catalog as well as the *Musa* Germplasm Information System (MGIS) database.

## Results

### Phylogenetic and blast identification analysis

**Fig. 1:**
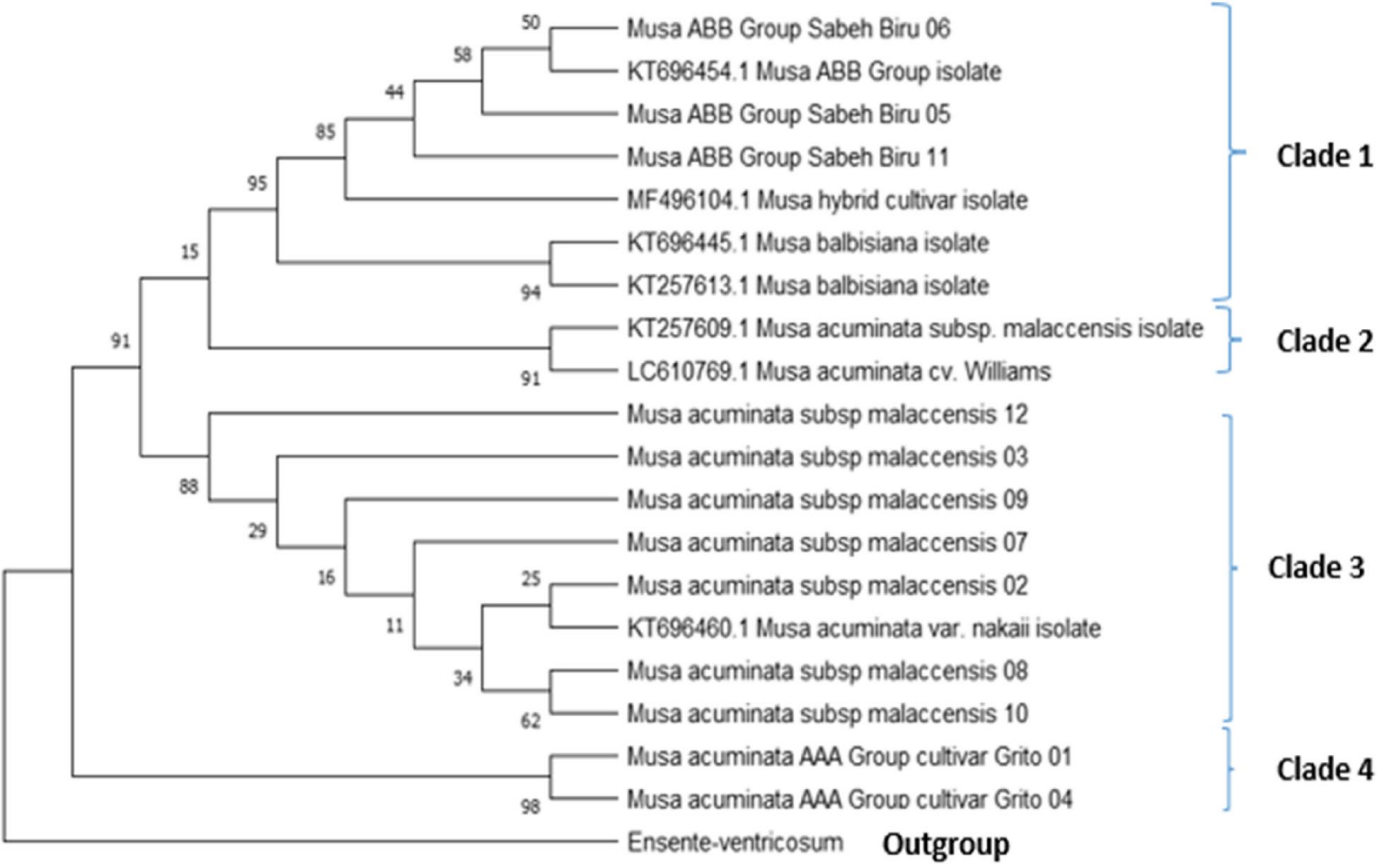
Evolutionary analysis of ITS1 sequences by maximum likelihood method*.* The evolutionary history was inferred by using the Maximum Likelihood method and Tamura-Nei model [20]. The tree with the highest log likelihood (− 10,841.64) is shown. The percentage of trees in which the associated taxa clustered together is shown next to the branches. Initial tree(s) for the heuristic search were obtained automatically by applying Neighbor-Join and BioNJ algorithms to a matrix of pairwise distances estimated using the Tamura-Nei model and then selecting the topology with superior log likelihood value. This analysis involved 20 nucleotide sequences. There were a total of 6810 positions in the final dataset. Evolutionary analyses were conducted in MEGA11 [22]

The phylogenetic tree for ITS 1 sequences resulted in four clades as shown in Fig. 1. The Clade 1 consists of 7 sequences, out of which 4 being retrieved from downloaded NCBI sequences. Blast inference identified the 3 sample sequences as Musa ABB Sabeh Biru cultivars. All sequences in this clade contain a B genome. The second clade consists of 2 sequences fetched from NCBI belonging to *Musa acuminata* species. Clade 3 consists of 8 sequences. The blast analysis revealed that 7 out of 8 of these sequences represent *Musa acuminata subsp malaccensis* while the 8th is from *Musa acuminata var. nakai* isolate. *Musa acuminata subsp malaccensis* sequence samples 12, 03, 09, and 07 showed more relationship distance from being *Musa acuminata var. nakai* isolate as compared to sample sequences 02, 08, and 10. Sample sequences 01 and 04 were identified as Musa acuminata AAA group cultivar *Grito* from the blast search and therefore made up the fourth clade. *Ensente ventricosum*, which belongs to the genera *Ensente* was the outgroup sequence.

**Fig. 2:**
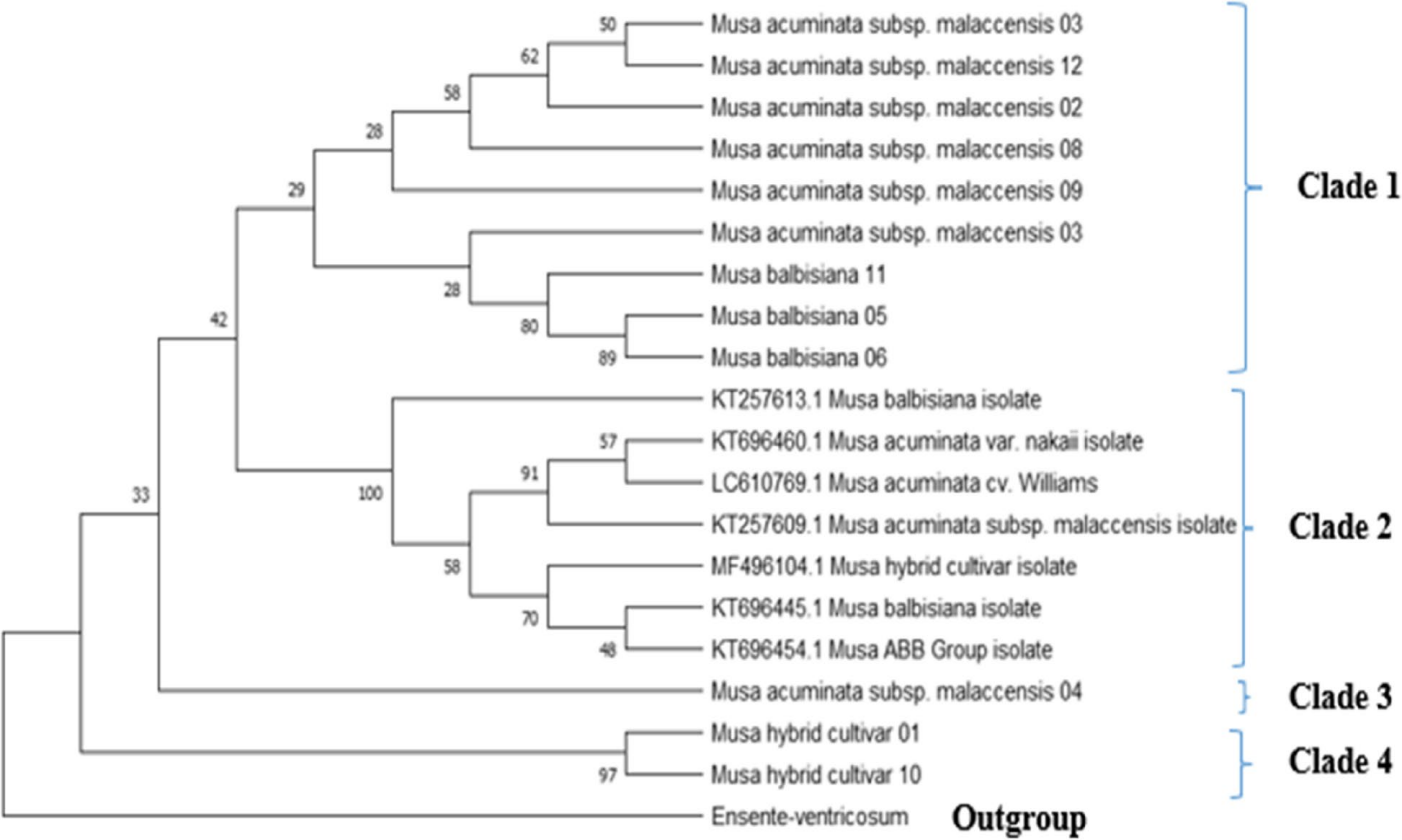
Evolutionary analysis of ITS4 sequences by maximum likelihood method. The evolutionary history was inferred by using the Maximum Likelihood method and Tamura-Nei model [20]. The tree with the highest log likelihood (− 14,146.13) is shown. The percentage of trees in which the associated taxa clustered together is shown next to the branches. Initial tree(s) for the heuristic search were obtained automatically by applying Neighbor-Join and BioNJ algorithms to a matrix of pairwise distances estimated using the Tamura-Nei model, and then selecting the topology with superior log likelihood value. This analysis involved 20 nucleotide sequences. There were a total of 6843 positions in the final dataset. Evolutionary analyses were conducted in MEGA11 [22]

The phylogenetic tree for ITS 4 sequences resulted in four clades as shown in Fig. 2. Clade 1 consisted of 9 sequences, 6 of them being blast inference *Musa acuminata subsp malancessis* species and the other three being blast inferred *Musa balbisiana* sequences. Clade 2 consisted of all NCBI downloaded sequences. The third clade comprises of only 1 sequence, a blast-identified sample sequence, *Musa acuminata subsp malaccensis*. Clade 4 consisted of 2 blat inferred Musa hybrid cultivars.

### Efficacy of ITS1 and ITS 4 for genetic identification

The average GC contents for ITS 1 and ITS 4 were shown to be 63.06% and 61.60% respectively https://jamiemcgowan.ie/bioinf/gc.html. ITS4 showed a range of 84.39–99.68% blast identification rate for the 12 *Musa* samples whilst ITS 1 showed an almost similar range of 84.32–99.84%. The length of the ITS 1 varied from 622 to 630 while those of ITS 4 ranged from 630 to 958 bp. With reference to Tables 1 and 2, there is a higher ambiguity percentage in identification using ITS 1 in sample sequences 01, 04, (which were identified as *Musa acuminata*) and also in samples 01, 04, 05, and 10 (*Musa* hybrid cultivar, *Musa* *acuminata* subsp. *Malaccensis*, *Musa balbisiana* and *Musa* AB hybrid respectively) in ITS 4 sequences. Sequences were identified to the cultivar level hence morphological identification was employed to name the local bananas as shown in Table 3.

**Table 3:** 1—Fig banana, 2—apple banana/*Nzarayapera*, 3—*Nzarayapera*, 4—williams, 5—giant Cavendish, 6—dwarf Cavendish, and 7—grand naine

Descriptor	*Musa* ABB	*Musa* AB hybrid	*Musa balbisiana*	*Musa acuminata*	*Musa acuminata malaccensis*
Bract					
Bract base shape	1—medium	2—medium	3—medium	4—Medium 5—Medium 6—Medium	7-Medium
Apex shape	Intermediate	Intermediate	Intermediate	4—Slightly pointed 5—Slightly pointed 6—Slightly pointed	Intermediate
Bract shape	Intermediate	Lanceolate	Intermediate	4—Intermediate 5—Intermediate 6—Intermediate	Intermediate
Bract lifting	Lifting 2/more	Lifting 2/more	Lifting 2/more	4—Lifting 2 /more 5—Lifting 2/more 6—	Lifting 2/more
Bract external color	Purple-brown	Red–purple	Orange-red	4—Pink-purple 5—Red purple 6—Deep purple	Purple
Behavior before falling	Revolute	Revolute	Revolute	4—Revolute 5—Revolute 6—Revolute	Revolute
Waxiness	Very waxy	Very waxy	Moderate	4—Very waxy 5—Moderate 6—Few wax	Very few wax
Male flower					
Dominant color	Pink-purple	yellow	Yellow	4—Yellow 5—Yellow 6—Yellow	yellow
Free Tepal color	Tinted pink	Tinted yellow	Tinted with yellow	4—Tinted yellow 5—Tinted yellow 6—Tinted yellow	Tinted yellow
Free tepal appearance	corrugated	corrugated	corrugated	4—Corrugated 5—Corrugated 6—Corrugated	Simple folding under the apex
Free tepal apex shape	Triangular	triangular	triangular	4—Oval 5—Triangular 6—Oval	Triangular
Inflorescence					
Male bud type	normal	normal	normal	4—Normal 5—Normal 6—Normal	Normal
Male bud shape	Intermediate	Intermediate	Intermediate	4—Intermediate 5—Intermediate 6—Intermediate	Intermediate
Bunch position	Vertical hang	slightly angled	Vertical hang	4—Vertical hang 5—Vertical hang 6—Vertical hang	Vertical hang
Bunch shape	Cylindrical	Cylindrical	Cylindrical	4—Cylindrical 5—Cylindrical 6—Cylindrical	cylindrical
Bunch appearance	Lax	Compact	Compact	4—Lax 5—Lax 6—Lax	lax
Rachis position	Falling vertically	Falling vertically	curvy	4—Angular 5—Angular 6—Falling vertical	curvy
Peduncle colour	Light green	Green	Light green	4—Light green 5—Green 6—Green	Light green
Fruit					
Shape	Straight in distal part	Straight	Straight	4—Slightly curvy 5—Curved 6—Curved	curved
Apex	Blunt tipped	Bottle-neck	Pointed	4—Blunt tipped 5—Bottle-necked 6—Blunt tipped	Blunt tipped
Pulp color at maturity	White	Yellow	Yellow	4—Cream 5—Cream 6—Cream	yellow
Days from planting to flowering	366	250	396	4—360 5—400 6—423	380
Days from flowering to harvest	135	100	145	4—120 5—118 6—135	137

### Morphological Characterization

**Fig. 3:**
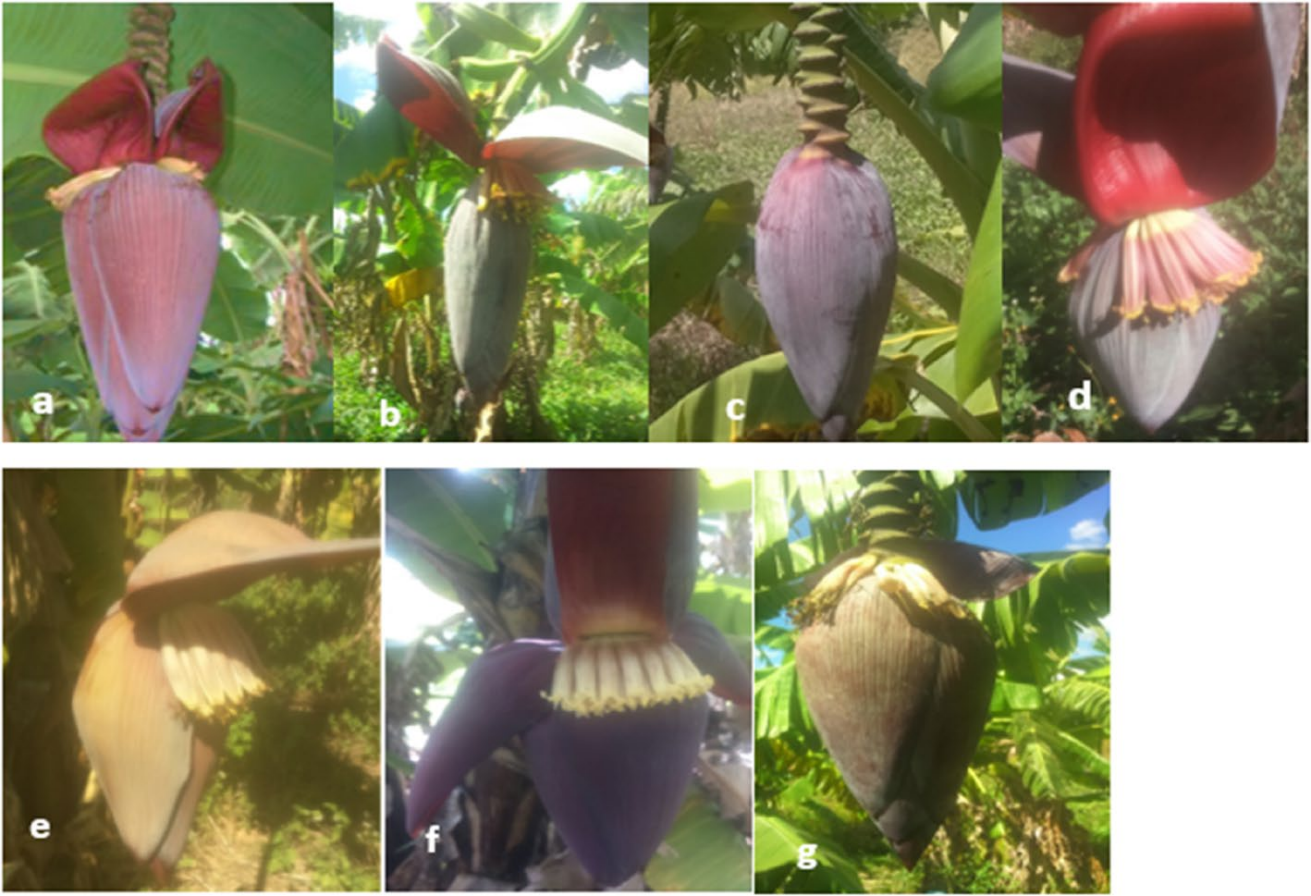
Inflorescence of banana cultivars from shown from image **a** to **g** where **a** shows Williams, **b** Fig, **c** *Nzarayapera*, **d** Grand naine, **e** Apple banana, **f** Dwarf Cavendish, **g** Giant cavendish

Morphological characterization of the cultivars was done focusing on the inflorescence as indicated in Fig 3. The bract, male flower and the fruits of the cultivars were characterized and matched against the IPB-INIBAP banana catalogue and tabulated as shown in Table 3. The descriptors used provided a distinct variation in the cultivars.

## Discussion

### Morphological analysis of identified cultivars

Characterization of the germplasm using morphological traits resulted in genomic groups being identified with international as well as local names. The *Musa* ABB cultivar was identified as a Fig banana type in this study, which is also known as the quince, *Sapa*, *Tanga,* or *Couruda* banana depending on the country. It is a hybrid of *Musa acuminata* and *balbisiana* and originates from the Philippines. The tree is a medium-sized plant that produced 20–35 plum straight, sweet fruits with cracks on their peels.

The *Musa* AB identified cultivar, also known as the apple banana is locally called ‘*Nzarayapera*’ which translates to ‘no-more hunger’. This is because the fruit is starchy and filling. This variety originated from Ecuador and is referred to as the *Manzano* banana, which is characterized by a sweet apple aroma hence its English name [10].

*Musa Balbisiana* cultivar was also identified as *Nzarayapera.* It is a starchy plantain that has tiny seeds at its center. The cultivar takes time to flower, unlike the *Musa AB* hybrid.

*Musa acuminata* identified cultivars were the Williams, Giant Cavendish, Dwarf Cavendish, and the Grand Naine. These cultivars are mostly grown for commercial purposes unlike the first three. The bract external color, male flower, and fruit apex give differentiation of one Cavendish from the other. Bracts, male bud shape, and mature fruit color allows the differentiation between ploidies and banana subgroups [23].

### Genetic evaluation analysis

Molecular identification has a higher ability to differentiate one cultivar from the other. The use of ITS in genetic diversity and phylogenetic studies showed a high level of interspecific divergence and has been used frequently. The study utilized maximum likelihood models to infer phylogeny based on the ITS1 and ITS4 regions. The sequences all clustered into 4 clades for both the ITS1-based tree (Fig. 1) and the ITS4-based tree (Fig. 2). In the present study, ITS1-5.8S–ITS2 region was found to have higher discriminatory ability in comparison with the ITS 4 region as it managed to accurately separate different genome types that possessed a close genetic resemblance [24, 25]. It can also be shown that the genetic resemblance is also shown in the phenotype of the 228 banana isolates.

Based on the BLAST results, the Fig banana of the ABB genome showed 99.6% similarity to the Sabeh Biruh cultivar. The apple banana was identified with two genomes using both ITS1 and ITS 4 sequences. It showed 88.6% AB hybrid genome whilst a 99% *Musa acuminata subsp. malaccensis* identical. *Musa balbisiana* commonly known as *Nzarayapera* showed a 99% similarity. Williams, giant Cavendish, and dwarf Cavendish of the AAA genome showed a higher similarity to the *Musa acuminata* Grito cultivar. Cultivar Grand naine showed a 99% identity to *Musa acuminata sub malaccensis*.

The distance-based identification system revealed that the *Musa balbisiana (Nzarayapera)* and the hybrid cultivars have similar Euclidean distance while *Musa acuminata* grand naine demonstrated the longest distance.

The results of the study show that the phylogeny of the *Musa* family is controversial but the discriminatory power of the ITS sequences gives conclusive evidence of distinct cultivars. However, there is a need for further identification and possibly barcoding of the cultivars [26–33].

## Conclusion

One of the major challenges in banana production and research is numerous cultivar names and synonyms in various languages hence reliable methods for species and cultivar identification are crucial for the conservation of germplasm. The study showed that the ITS sequencing coupled with analysis of morphological traits is a useful tool in the characterization of bananas. The ITS1-5.8S–ITS2 region was found to have higher discriminatory ability in comparison with the ITS 4 region. It allowed genetic distinction of two locally grown *Nzarayapera* cultivars as well as allowing the identification of commercially grown varieties giving the country a guide in banana farming.

## Data Availability

All data generated or analyzed during this study are included in this published article and supplementary material.
